# A Novel Approach to Early Personalized Hemodynamic Resuscitation: Non‐Invasive Peripheral Photoplethysmography for Identifying Predominant Vasodilatory Shock in Sepsis

**DOI:** 10.1111/aas.70119

**Published:** 2025-09-09

**Authors:** Sanne Ter Horst, Anna D. Schoonhoven, Raymond J. van Wijk, Rick Weitering, Sanne W. van Loon, Jan C. ter Maaten, Hjalmar R. Bouma

**Affiliations:** ^1^ Department of Internal Medicine University of Groningen, University Medical Center Groningen Groningen the Netherlands; ^2^ Department of Acute Care University of Groningen, University Medical Center Groningen Groningen the Netherlands; ^3^ Department of Clinical Pharmacy and Pharmacology University of Groningen, University Medical Center Groningen Groningen the Netherlands

**Keywords:** critical care, photoplethysmography, resuscitation, sepsis, shock

## Abstract

**Introduction:**

Sepsis remains a leading cause of mortality, with mortality from septic shock exceeding 40%. Standardized resuscitation (30 mL/kg) may cause adverse outcomes, including fluid overload or prolonged hypotension, emphasizing the need for individualized strategies. Sepsis‐induced shock arises from varying degrees of vasodilation and hypovolemia, yet patients often present with similar clinical signs in the emergency department (ED). Photoplethysmography (PPG), a non‐invasive technique reflecting peripheral perfusion, may help identify patients with a predominant vasodilatory profile who could benefit from early vasopressor therapy.

**Methods:**

This post hoc analysis used data from the Acutelines biobank at the University Medical Centre Groningen. Adults admitted for non‐trauma specialties with suspected infection and hemodynamic instability (MAP < 70 mmHg, SBP < 90 mmHg, shock index > 0.9, or lactate > 4.0 mmol/L) were included. PPG data were pre‐processed and features extracted. Principal component analysis (PCA) and K‐means clustering enabled dimensionality reduction and hemodynamic profiling. Logistic regression assessed the discriminative performance of PPG‐based models for vasopressor therapy initiation within 24 h.

**Results:**

Among 325 patients, 16.3% received vasopressors. PCA identified three principal components explaining 80.3% of variance: PC1 (arterial compliance), PC2 (cardiac output and systemic vascular resistance), and PC3 (peripheral vasomotor tone). The PPG‐based model showed moderate discriminative power (AUROC: 0.75), improving when combined with MAP and lactate (AUROC: 0.83).

**Conclusion:**

PPG enables identification of patients likely to benefit from vasopressor therapy during the first 20 min after ED arrival. By providing additional insight into peripheral perfusion, this proof‐of‐principle study supports further exploration of PPG as a clinical support tool for personalized hemodynamic resuscitation in sepsis.

**Editorial Comment:**

This secondary analysis demonstrates early peripheral circulatory patterns in sepsis using photoplethysmography at the start of resuscitation. Distinct PPG‐derived profiles were associated with vasopressor initiation within 24 h, supporting PPG as a tool for personalized resuscitation.

**Abbreviations**
AC: alternating current
APG: acceleration photoplethysmogram
AU: arbitrary units
AUROC: area under the receiver operating characteristic
CMV: cytomegalovirus
CO: cardiac output
COPD: chronic obstructive pulmonary disease
CT: crest time
CVD: cardiovascular disease
DC: direct current
DPA: diastolic peak amplitude
DT: delta time
ECG: electrocardiography
ED: emergency department
GCS: glasgow coma scale
HR: heart rate
ICU: intensive care unit
IPA: inflection point area
IQR: interquartile range
IR: infrared
IRB: institutional review board
I.V.: intravenous
MAP: mean arterial pressure
NEWS: national early warning score
NPV: negative predictive value
PCA: principal component analysis
PC: principal component
PI: pulse interval
POI: point‐of‐interest
PPG: photoplethysmography
PPI: perfusion index
PPV: positive predictive value
PW: pulse width
qSOFA: quick sequential related organ failure assessment
REDcap: research electronic data capture
RI: reflection index
ROC: receiver operating characteristic
SBP: systolic blood pressure
SOFA: sequential organ failure assessment
SPA: systolic peak amplitude
SQI: signal quality index
SVR: systemic vascular resistance
UMCG: university medical center groningen
WHO: world health organization

## 1 Introduction

Sepsis is the leading global cause of mortality, with a mortality rate of 10% that increases to more than 40% in the case of septic shock [1, 2]. In 2017, the World Health Organization [WHO] estimated that sepsis affected 49 million people globally, resulting in 11 million deaths worldwide [3]. Sepsis is defined as a life‐threatening organ dysfunction caused by a dysregulated host response a reaction to infection, culminating in organ failure and shock [2]. Early treatment of hemodynamic instability in a patient with sepsis is of utmost importance to prevent organ failure and death.

Current sepsis treatment includes timely antibiotics and hemodynamic resuscitation with intravenous fluids and, when needed, vasopressor therapy [1, 4]. The Surviving Sepsis Campaign Guidelines still recommend administering at least 30 mL/kg of crystalloid fluids for patients with sepsis‐induced hypoperfusion or septic shock to restore organ perfusion [4, 5]. Vasopressors are indicated in cases of persistent hypotension despite adequate fluid resuscitation, or when fluids are either unlikely to restore hemodynamic stability or contraindicated, targeting a mean arterial pressure (MAP) of ≥ 65 mmHg [6]. However, a one‐size‐fits‐all approach is problematic due to significant inter‐individual differences in fluid responsiveness and tolerance [7, 8]. Only about half of patients are fluid responsive; others risk fluid overload [9, 10, 11], which can lead to venous congestion, worsened organ dysfunction, prolonged intensive care unit (ICU) stays, and increased mortality [12, 13, 14, 15]. Notably, signs of venous congestion have been observed in both fluid responsive and unresponsive critically ill patients, underscoring the complexity of fluid management [10]. Emerging evidence suggests that very early vasopressor support seems safe, may reduce the total volume of fluids required, and thereby could improve clinical outcomes [16, 17]. These findings emphasize the need for a personalized approach to hemodynamic resuscitation, carefully balancing the choice between higher‐volume fluid resuscitation or early initiation of vasopressor therapy to optimize patient outcomes [9, 18].

A personalized strategy based on patient‐specific cardiovascular changes is essential to optimize resuscitation, prevent organ failure, and potentially reduce sepsis‐related morbidity and mortality. Septic shock typically involves both vasodilatory and hypovolemic components, with some patients also experiencing cardiogenic shock due to septic cardiomyopathy [18, 19, 20]. Vasodilatory shock, marked by excessive vasodilation and low systemic vascular resistance, requires vasopressors to restore vascular tone [21, 22]. Hypovolemic shock can be absolute, caused by fluid loss from vomiting, diarrhea, or bleeding, or relative, resulting from venous vasodilation and increased venous capacitance that reduce venous return [22, 23]. While absolute hypovolemia primarily requires fluid administration, relative hypovolemia may benefit from both fluids and vasopressors to improve venous return and maintain perfusion [23, 24]. Complete differentiation between these mechanisms is often not possible, but identifying the predominant physiological driver can help guide early and effective management. Therefore, incorporating cardiovascular parameters that reflect these pathophysiological changes could improve individualized treatment decisions and help identify sepsis patients who would benefit from early vasopressor therapy.

Photoplethysmography (PPG) is a non‐invasive optical technique commonly integrated in pulse oximeters [25]. While pulse oximetry primarily measures oxygen saturation, PPG waveform analysis provides additional insight by reflecting peripheral blood volume changes [25, 26], which are influenced by cardiac output, arterial compliance, systemic vascular resistance, and vasomotor tone [27, 28]. We hypothesize that PPG‐derived features reflect the hemodynamic effects of sepsis, particularly peripheral vasodilation, enabling identification of patients who might benefit from early vasopressor therapy in the emergency department (ED). Therefore, in this proof‐of‐principle study, we first aimed to identify early septic shock subphenotypes based on PPG‐derived features. Second, we explored whether PPG measured in the first 20 min of ED triage could identify individual patients with a predominant vasodilatory shock component, as defined by receiving vasopressor therapy within 24 h, who may potentially benefit from early vasopressor intervention.

## 2 Methods

### 2.1 Study Design

We conducted a post hoc analysis of prospectively obtained data by Acutelines: a data‐, image‐, and bio‐bank at the ED of the University Medical Center Groningen (UMCG) [19]. A deferred consent procedure (by proxy) was in place to enable the collection of data and biomaterials before obtaining written consent. When reaching the patient or proxy was not feasible, we followed an opt‐out procedure. Acutelines is approved by the medical ethics board of the UMCG and registered under trial registration number NCT04615065 at ClinicalTrials.gov. The study protocol was approved by the institutional review board under registration number 18089. Acutelines' complete protocol and overview of the actual, entire data dictionary is available via www.acutelines.nl [29].

### 2.2 Study Population

The inclusion criteria for this study were: patients with an age above 18 years (i), admitted for either internal medicine, rheumatology, gastro‐enterology, pulmonology, or emergency medicine for a non‐traumatic reason (ii), who had a suspected infection based on physicians' discretion at ED arrival (iii), in need of hemodynamic resuscitation based on MAP < 70 mmHg, systolic blood pressure (SBP) < 90 mmHg, shock index (heart rate (HR)/SBP) > 0.9, or lactate > 4.0 mmol/L, and (iv) had available PPG waveform data captured by the Philips bed‐side monitor (v). The exclusion criteria were: patients with a non‐ICU admission policy outlined in an advanced care directive (i), and absent or low‐quality PPG data according to the determined signal quality index (SQI) described in the section below (ii). For the main study cohort, we included all eligible patients between September 2020 and December 2023. For the internal validation cohort, we included all eligible patients between January 2024 and June 2024.

### 2.3 Data Capture and Definitions

To allow collection of data and biomaterials upon first contact, primary screening of patients for eligibility upon arrival in the ED was performed 24/7 by the ED‐nurse with a trained research team. Continuous bedside physiological waveforms (e.g., electrocardiography (ECG), PPG, and impedance pneumography) and vital parameters were automatically captured by a Philips IntelliVue MP70 or MX550 with a multi‐measurement module bedside monitor in the ED and securely stored on a network drive for later analysis (Sept 2020–Dec 2021, Apr 2022–July 2022, and Jan 2023–June 2024). Information from other data sources such as the hospital's electronic health records was securely imported. Study data was collected using REDCap electronic data capture tools hosted at the UMCG [30]. For the main study cohort, we included all PPG and clinical data, including primary and secondary endpoints. For the internal validation cohort, we collected PPG data, age, gender, cardiovascular comorbidities, intravenous (I.V.) fluids, vital signs, and the use of vasopressor therapy < 24 h. For this study, we used the first 20 min of PPG waveform recorded after arrival at the ED, which matches the moment of triage when the patient's clinical condition is typically assessed based on vital parameters. Infection foci were confirmed post hoc based on the assessment of an expert adjudication panel.

### 2.4 Outcomes

The primary endpoint in this study was the initiation of vasopressor therapy within 24 h after ED arrival. The secondary endpoint in this study was the administration of > 30 mL/kg intravenous fluids within the first 3 h in the ED. Additional endpoints included ICU admission and in‐hospital mortality within 48 h.

### 2.5 Waveform Pre‐Processing, Feature Extraction and Characterization

**FIGURE 1:**
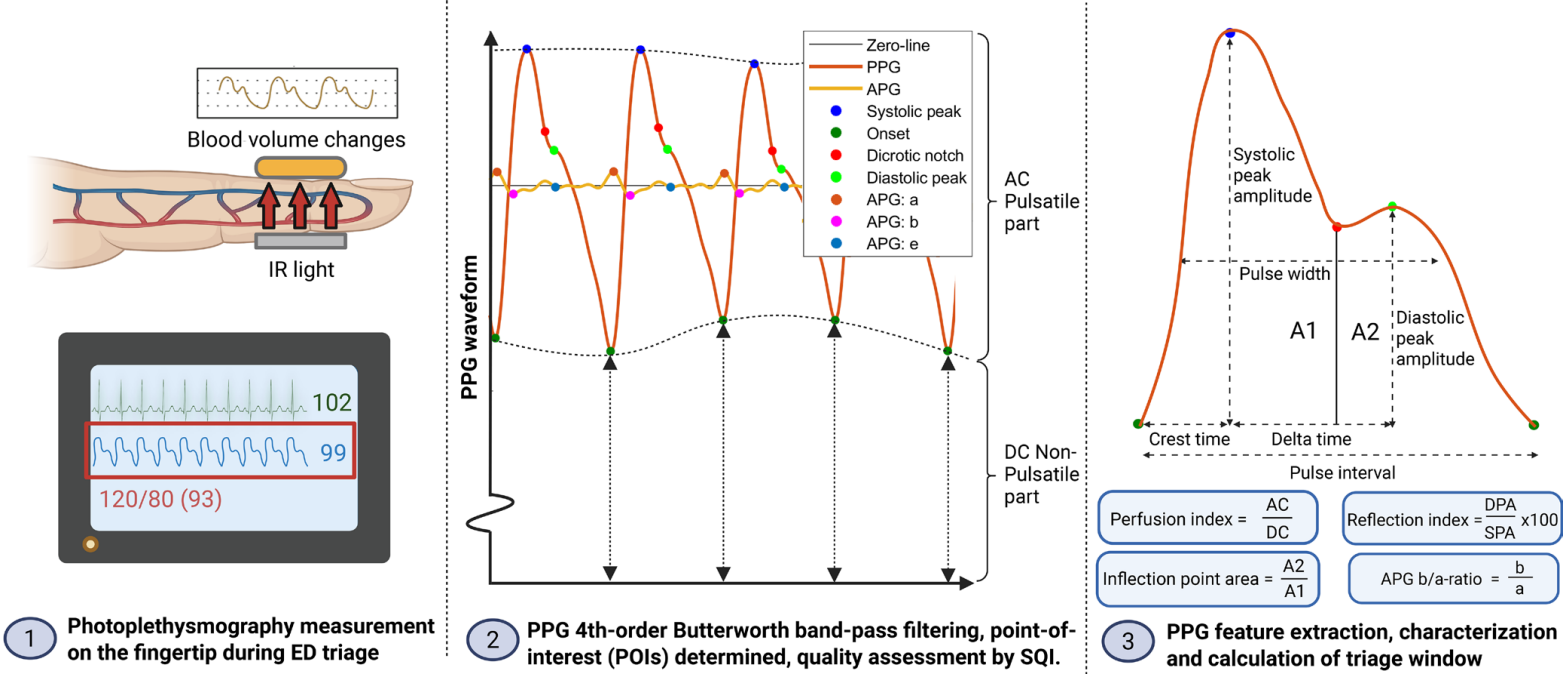
Schematic outline of PPG waveform measurement, preprocessing, feature extraction, and characterization. (1) Pulse oximetry measurement during ED triage—A pulse oximeter is placed on the patient's fingertip during ED triage to obtain the photoplethysmogram (PPG) waveform. (2) PPG waveform preprocessing—The raw PPG waveform is filtered using a 4th‐order Butterworth band‐pass filter (0.8–20 Hz) to isolate the relevant signal. Both the pulsatile (AC) and non‐pulsatile (DC) components are retained, as the latter is used to determine the perfusion index. Points‐of‐interest (POIs) are identified within the PPG waveform, including the systolic peak, wave onset, dicrotic notch, diastolic peak, a‐peak, b‐peak, and e‐peak. PPG waves with low correlation to an averaged template are excluded. (3) PPG feature extraction and characterization—Following preprocessing and quality assessment, features are extracted from both the PPG waveform (pulsatile (AC) and non‐pulsatile (DC)) and its second derivative, the acceleration photoplethysmogram (APG). These features are then averaged over the first 20 min for analysis. A1, Area 1; A2, Area 2; AC, alternating current; APG, acceleration photoplethysmogram; DC, direct current; DPA, diastolic peak amplitude; ED, emergency department; IR, Infrared; PPG, pulse oximetry photoplethysmography/g; SPA, systolic peak amplitude; Created with Biorender.com.

First, we pre‐processed the continuous raw PPG waveform using MATLAB R2018a (Matlab, the Mathworks, Natick, USA). The initial pre‐processing step involved filtering using a 4th‐order Butterworth band‐pass filter with a frequency range of 0.8 to 20 Hz. The next step involved evaluating the patient's PPG waveform data quality for feature extraction. This assessment was performed by calculating the Signal Quality Index (SQI) [31]. To this end, we first identified the points‐of‐interest (POIs) within the raw PPG waveform, which encompassed the systolic peak, wave onset, dicrotic notch/inflection point, diastolic peak, a‐peak, b‐peak, and e‐peak (Figure 1). Next, a correlation coefficient was computed for each PPG wave by comparing it to an averaged wave template representing the average wave for the specific patient. PPG waves with a correlation coefficient lower than 0.8 were removed from the analysis. This signal was then split into one‐minute windows, resulting in 20 windows per patient in total. For further SQI determination, a one‐minute window was excluded when it contained less than five PPG waves. PPG data was considered low‐quality when it contained less than two 2 min windows in a 20 min time frame. Following this exclusion process, PPG‐derived features were computed per 1 min window, using the identified POIs (Figure 1). After feature extraction, the mean values were calculated over the initial 20 min. For our analysis, we extracted specific features from the PPG waveform itself and its second derivative, the acceleration photoplethysmogram (APG; Table S1 and Figure 1) [27, 28, 32, 33, 34].

### 2.6 Statistical Analysis

Continuous data are presented as medians with interquartile ranges (IQRs), and categorical data as counts and percentages (*n*, %). Data distribution normality was assessed using the Shapiro–Wilk test. For non‐normally distributed data, the Mann–Whitney *U* test was used for pairwise comparisons, and the Chi‐square test was used for categorical variables. For comparisons across three groups, the Kruskal‑Wallis test was applied for continuous variables, and the Fisher's exact test was used for categorical variables. PPG‐derived features were scaled and integrated using principal component analysis (PCA) to reduce dimensionality, address collinearity, and capture the majority of variance. Principal components that together explained more than 70% of the total variance were retained for further analysis. A correlation matrix was used to explore relationships between individual PPG‐derived features and principal components. Subsequently, the analysis was then divided into two complementary approaches. First, unsupervised *K*‐means clustering was applied to the retained principal components to explore whether early septic shock subphenotypes with distinct PPG patterns could be identified. The clinical and hemodynamic characteristics of patients within each cluster were subsequently examined. Second, we conducted supervised multivariable logistic regression models to evaluate the discriminative ability of PPG‐derived components in distinguishing patients who did or did not receive vasopressor therapy within 24 h, as well as other clinical outcomes. Independent variables included PPG‐derived principal components, MAP, and lactate. These models were adjusted for known confounders (e.g., age, gender, pre‐hospital I.V. fluids, cardiovascular comorbidities) and assessed using the Area Under the Receiver Operating Characteristic (AUROC) curve. To assess the diagnostic accuracy of PPG as compared to current parameters, we developed multiple models that integrated MAP, lactate, and PPG in different combinations. The robustness of the PPG model was further evaluated through internal cohort validation (2024). Statistical analyses were performed using RStudio (version 4.3.1), and figures were created using GraphPad Prism 10, Biorender.com, and Adobe Illustrator 2024.

## 3 Results

### 3.1 Study Population

**FIGURE 2:**
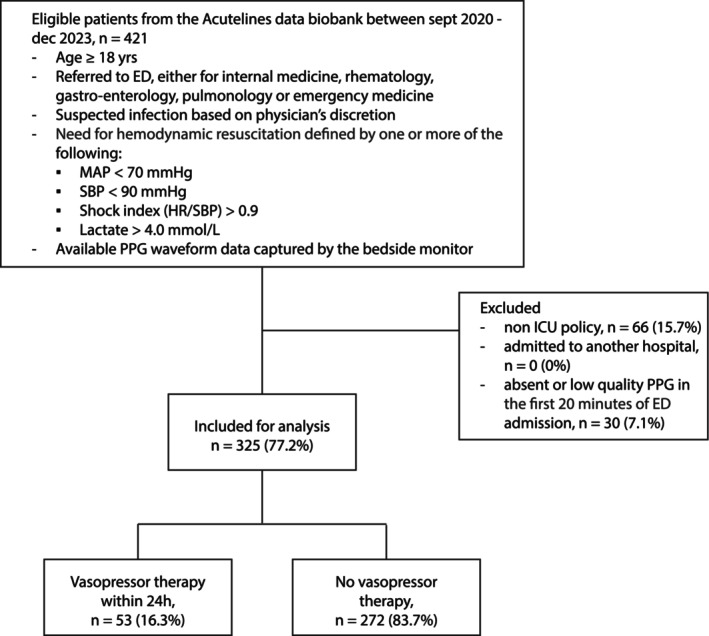
Flowchart of patient selection. ED, emergency department; HR, heart rate; ICU, intensive care unit; MAP, mean arterial pressure; PPG, pulse oximetry photoplethysmography; SBP, systolic blood pressure.

We included 325 of 421 eligible patients (77%) from the Acutelines data biobank who were admitted to the ED with a working diagnosis of sepsis requiring hemodynamic resuscitation and available PPG waveforms for analysis (Figure 2). The remaining 96 patients (23%) were excluded for the following reasons: 66 patients (16%) were excluded due to a non‐ICU treatment policy, which meant no use of vasopressors, and 30 patients (7%) were excluded due to absent or low‐quality PPG waveforms in the first 20 min. The cohort was 41% female, with a median age of 63, IQR: [50–73]. Relevant comorbidities included 120 patients (37%) with hypertension, 34 (11%) with ischemic heart disease, 41 (13%) with heart failure, and 90 (28%) with diabetes. The median sequential organ failure assessment (SOFA) score was 4 [2, 3, 4, 5, 6, 7]. In terms of hemodynamic management, patients received a median pre‐hospital of 0.5 [0.0, 0.5] L of I.V. fluids, and in ED a median 0.6 [0.1, 1.5] L of I.V. fluids, with 63 (19.4%) receiving more than 30 mL/kg/3 h. Among all patients, 53 (16%) received vasopressor therapy within 24 h, while 272 (84%) did not. Additionally, 82 (25%) patients were admitted to the ICU within 48 h, and 14 (4%) died within 48 h. The diagnosis of an infection was confirmed by a post hoc adjudication committee: 107 patients (33%) had a respiratory focused infection, 48 (15%) had a urinary tract infection, 87 (27%) had other sites of infection, and 83 (26%) had no confirmed infection (Table 1).

**TABLE 1:** Characteristics of study population.

Characteristics of the study cohort	*N* = 325
Demographics	
Female (*n* (%))	132 (41)
Age (median [IQR])	63 [50, 73]
Co‐morbidities	
Charlson Comorbidity Index (median [IQR])	4 [2, 6]
Hypertension (*n* (%))	120 (37)
Ischemic heart disease (*n* (%))	34 (11)
Heart failure (*n* (%))	41 (13)
Diabetes (*n* (%))	90 (28)
Asthma or COPD (*n* (%))	50 (15)
Chronic liver disease (*n* (%))	11 (3)
Chronic kidney disease (*n* (%))	32 (10)
Transplantation (*n* (%))	35 (11)
Malignancy (*n* (%))	75 (23)
Hematologic malignancy (*n* (%))	37 (11)
Vital parameters at triage	
Systolic blood pressure [mmHg] (median [IQR])	105 [91, 121]
Diastolic blood pressure [mmHg] (median [IQR])	65 [53, 78]
Mean arterial blood pressure [mmHg] (median [IQR])	80 [65, 91]
Heart rate [beats/min] (median [IQR])	113 [96, 126]
Respiratory frequency [/min] (median [IQR])	22 [18, 26]
Oxygen Saturation (SpO2) [%] (median [IQR])	97 [94, 98]
Temperature (°C) (median [IQR])	37.1 [36.4, 38.1]
GCS (median [IQR])	15 [15, 15]
Lab values at triage	
Blood gas lactate (mmol/L) (median [IQR])	2.8 [2.1, 4.4]
Leukocytes (×10^9^/L) (median [IQR])	12.4 [7.2, 17.2]
Trombocytes (×10^9^/L) (median [IQR])	205 [148, 282]
C‐reactive protein (mg/L) (median [IQR])	73 [20, 173]
Creatinine (umol/L) (median [IQR])	110 [78, 171]
Bilirubin (umol/L) (median [IQR])	11 [7, 18]
Scoring systems at ED triage	
qSOFA (median [IQR])	1 [1, 2]
SOFA (median [IQR])	4 [2, 7]
NEWS (median [IQR])	6 [4, 9]
Hemodynamic support therapy	
Prehospital I.V. fluids (i.e., in ambulance) (L) (median [IQR])	0.5 [0.0, 0.5]
ED I.V. fluids (L) (median [IQR])	0.6 [0.1, 1.5]
Resuscitation according to 30 mL/kg/3 h (*n* (%))	63 (19)
Vasopressor therapy (i.e., noradrenaline) < 24 h (*n* (%))	53 (16)
Clinical deterioration outcomes	
ICU admission < 48 h (*n* (%))	82 (25)
In‐hospital mortality < 48 h	14 (4)
In‐hospital mortality < 7 days	29 (9)
Mortality < 30 days	48 (15)
Infection foci confirmed by adjudication panel	
Intra‐abdominal infection	26 (8)
Gastro enteritis	31 (10)
Respiratory tract infection	107 (33)
Urinary tract infection	48 (15)
Endocarditis/pericarditis	1 (1)
Skin/joint infection	16 (5)
Viral other infection (i.e., herpes zoster, CMV)	3 (1)
Infection eci (i.e., unknown focus)	10 (3)
No confirmed infection	83 (26)

*Note:* The table shows median and interquartile ranges (IQR) between brackets for continuous variables and absolute number and percentages for categorical variables.

Abbreviations: COPD, chronic obstructive pulmonary disease; GCS, Glasgow Coma Scale; IQR, interquartile range; NEWS, national early warning score; SOFA, sequential organ failure assessment; qSOFA, quick sequential organ failure assessment.

Following post hoc infection adjudication, we compared baseline characteristics, vital signs, and PPG‐derived features between patients with confirmed infection and those without (Table S1). The confirmed infection group showed lower pulse width (PW), reflection index (RI), and inflection point area (IPA), indicating increased vasodilation, and a shorter pulse interval (PI), consistent with tachycardia. Lactate was higher in the no‐infection group, possibly due to other acute aetiologies such as diabetic ketoacidosis. Despite fewer I.V. fluids, their outcomes were similar. As clinical decisions rely on infection suspicion at ED arrival, all patients were included in the main analysis to reflect real‐world practice.

### 3.2 Principal Component Analysis and Clustering Reveal Hemodynamic Profiles

**FIGURE 3:**
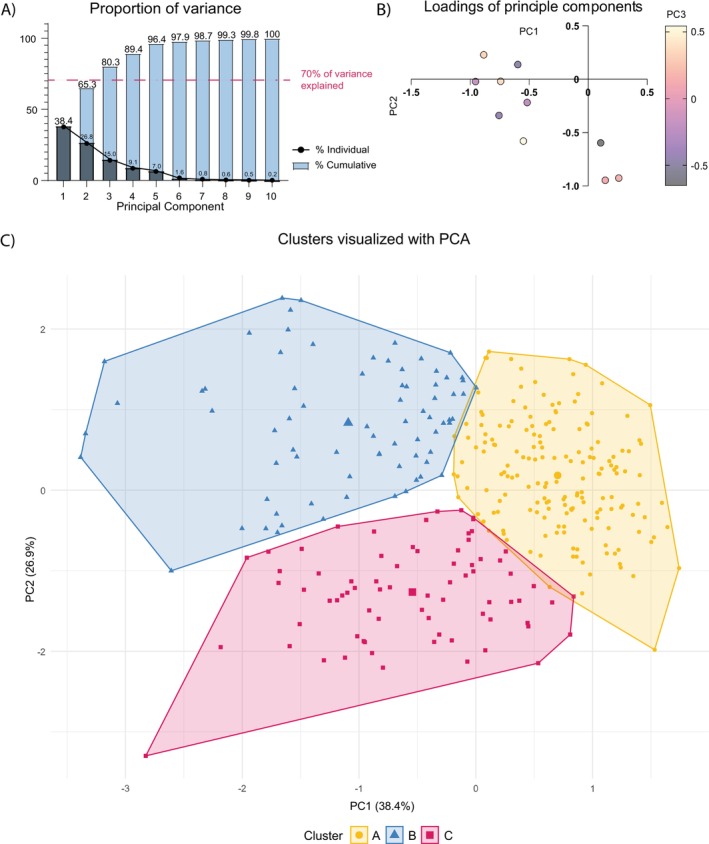
Principal component analysis and *K*‐means clustering. (A) The proportion of variance explained by each principal component, with the pink dotted line indicating the 70% cumulative variance threshold. The plot presents both the individual and cumulative variance explained by each principal component (%). (B) A plot of the loadings for each PPG feature, where the loading of PC1 is on the *y*‐axis, the loading of PC2 is on the *x*‐axis, and the loading of PC3 is represented by the color scale (light = positive, dark = negative). This visualization illustrates the relative contribution of each PPG feature to the first three principal components. (C) Results of *K*‐means clustering based on the first three principal components. A two‐dimensional representation of PC1 and PC2 is shown, which together explain the majority of the variance in the data.

After preprocessing, PPG‐derived features were scaled and analyzed using principal component analysis (PCA), with the first three components explaining 80.3% of the variance (PC loadings in Table 2 and Figure 3). PC1 was primarily characterized by PW (−0.487) and RI (−0.451), reflecting arterial compliance. PC2 was mainly characterized by Systolic Peak Amplitude (SPA, 0.566) and Perfusion Index (PPI, 0.578), which relate to cardiac output (CO) and systemic vascular resistance (SVR). PC3 was largely shaped by Diastolic Peak Amplitude (DPA, 0.442) and Delta Time (DT, −0.530), reflecting peripheral vasomotor tone, associated with vasodilation in sepsis. Correlations between PPG‐derived features and principal components were consistent with PC loadings. PC1 correlated strongly with PW (*ρ* = 0.95), IPA (*ρ* = 0.80), PI (*ρ* = 0.72), and RI (*ρ* = 0.92); PC2 with SPA (*ρ* = 0.94) and PPI (*ρ* = 0.96); and PC3 with DPA (*ρ* = 0.56) and DT (*ρ* = 0.59; Figure S1).

**TABLE 2:** PPG feature loadings and explained variance in the first three principal components.

Feature	PC1	Variance explained within PC1 (%)	PC2	Variance explained within PC2 (%)	PC3	Variance explained within PC3 (%)
SPA	0.132	1.8	**0.566**	**32.0**	0.142	2.0
DPA	−0.280	7.9	0.354	12.5	**0.442**	**19.5**
CT	−0.304	9.3	−0.084	0.7	−0.381	14.6
DT	0.054	0.3	0.364	13.3	**−0.530**	**28.1**
IPA	−0.379	14.3	0.012	< 0.1	0.301	9.1
PI	−0.386	14.9	0.207	4.3	−0.359	12.9
PW	**−0.487**	**23.7**	0.014	< 0.1	−0.138	1.9
RI	**−0.452**	**20.4**	−0.140	2.0	0.281	7.9
APG	−0.264	7.0	0.133	1.8	−0.171	2.9
PPI	0.074	0.5	**0.578**	**33.5**	0.102	1.1

*Note:* The table presents the loadings of each variable on the principal components (PCs) identified through PCA. Loadings indicate the correlation between each variable and the PCs, with values greater than 0.400 or less than −0.400 highlighted in bold to emphasize their strong contributions. Variables with the highest absolute loadings on a PC are the most influential in defining that component. Additionally, the variance explained by each feature in the respective principal component is included in the table; PC1 (total explained variance = 38.4%): Dominated by pulse width (PW) and Reflection Index (RI), which contribute the most to this component, potentially reflecting shared physiological processes related to arterial compliance; PC2 (total explained variance = 26.9%): Strongly influenced by systolic peak amplitude (SPA) and Perfusion Index (PPI), which may relate to cardiac output and systemic vascular resistance; PC3 (total explained variance = 15.0%): Primarily characterized by diastolic peak amplitude (DPA) and delta time (DT), potentially capturing hemodynamic patterns associated with vasomotor tone.

**FIGURE 4:**
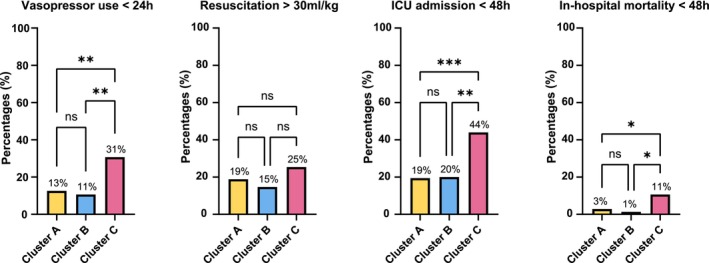
Distribution of study endpoints across PC clusters. These bar plots showing the distribution of the primary endpoint, that is, vasopressor use within 24 h, and secondary endpoints, including fluid resuscitation > 30 mL/kg during ED admission, ICU admission within 48 h, and in‐hospital mortality within 48 h after ED presentation, across the 3 PC clusters. Statistical significance of these differences between clusters was assessed using a chi‐squared test. * < 0.05 ** < 0.01 *** < 0.001; ns, not significant.

Unsupervised *K*‐means clustering of the three principal components identified three distinct clusters, with patient demographics, comorbidities, and clinical characteristics summarized in Table 3. Cluster C exhibited the most severe clinical profile, with lower blood pressure, higher heart rate, elevated respiratory frequency, and lower temperature. These patients also had elevated blood gas lactate, leukocyte, and creatinine levels, as well as the highest clinical severity scores (Table 3). Moreover, patients in Cluster C had the worst outcomes, including the highest rates of vasopressor therapy initiation within 24, 48 ICU admission, and 48 h mortality (Figure 4). In contrast, both Cluster A and Cluster B represented patients with early hemodynamic instability with better clinical outcomes than Cluster C. Despite these differences, the volume of intravenous fluid resuscitation during ED admission did not differ significantly between clusters (Figure 4). In summary, Cluster C appears more severely ill and in greater need of hemodynamic support (i.e., vasopressor therapy, ICU admission) despite similar fluid volumes, suggesting these patients may be experiencing refractory vasodilatory shock rather than hypovolemic shock, with instability driven by factors like vasodilation due to disturbed vasomotor tone in sepsis. A limited subgroup analysis comparing patients with no usable PPG signal to those in Cluster C showed comparable clinical characteristics (Table S2), suggesting that absent or poor‐quality PPG may also indicate underlying hemodynamic compromise.

**TABLE 3:** Characteristics of study population per PC cluster.

Characteristics	Cluster A	Cluster B	Cluster C	*p*
*N* (%)	175 (54)	75 (23)	75 (23)	
Demographics				
Female (*n* (%))	74 (42)	32 (43)	26 (35)	0.488
Age (median [IQR])	59 [46, 68]^B,C^	67 [54, 75]^A^	70 [57, 77]^A^	**< 0.001**
Co‐morbidities				
Charlson Comorbidity Index (median [IQR])	3 [2, 6]	4 [1, 6]	4 [3, 7]	0.071
Ischemic heart disease (*n* (%))	15 (9)	10 (13)	9 (12)	0.468
Heart failure (*n* (%))	16 (9)^B^	15 (20)^A^	10 (13)	0.059
Hypertension (*n* (%))	51 (29)^B,C^	34 (45)^A^	35 (47)^A^	**0.007**
Diabetes (*n* (%))	52 (30)	21 (28)	17 (23)	0.520
Asthma or COPD (*n* (%))	24 (14)	9 (12)	17 (23)	0.129
Chronic liver disease (*n* (%))	8 (5)	2 (3)	1 (1)	0.399
Chronic kidney disease (*n* (%))	18 (10)	7 (9)	7 (9)	0.960
Transplantation (*n* (%))	17 (10)	12 (16)	6 (8)	0.230
Malignancy (*n* (%))	43 (25)	12 (16)	20 (27)	0.237
Hematologic malignancy (*n* (%))	22 (13)	8 (11)	7 (9)	0.743
Vital parameters at triage				
Systolic blood pressure [mmHg] (median [IQR])	109 [95, 123]^C^	104 [90, 117]	99 [84, 115]^A^	**0.005**
Diastolic blood pressure [mmHg] (median [IQR])	66 [55, 78]	61 [50, 80]	61 [51, 76]	0.115
Mean arterial blood pressure [mmHg] (median [IQR])	81.7 [70.2, 91.2]^C^	74.67 [63.7, 94.8]	73.7 [62.8, 86.8]^A^	**0.027**
Heart rate [beats/min] (median [IQR])	120 [106, 133]^B,C^	96 [78, 112]^A,C^	111 [95, 121]^A,B^	**< 0.001**
Respiratory frequency [/min] (median [IQR])	22 [18, 26]^B^	20 [16, 23]	23 [20, 27]^B^	**0.002**
Oxygen Saturation (SpO2) [%] (median [IQR])	96 [95, 98]	97 [94, 99]	97 [94, 99]	0.331
Temperature (°C) (median [IQR])	37.5 [36.7, 38.4]^B,C^	36.7 [36.0, 37.5]^A^	36.6 [36.1, 37.4]^A^	**< 0.001**
GCS (median [IQR])	15 [15, 15]	15 [15, 15]	15 [15, 15]	0.576
Lab values at triage				
Blood gas lactate (mmol/L) (median [IQR])	2.6 [2.1, 4.4]^C^	2.6 [1.5, 4.0]^C^	3.8 [2.8, 5.2]^A,B^	**< 0.001**
Leukocytes (×10^9^/L) (median [IQR])	11.0 [6.1, 15.9]^C^	13.6 [8.7, 17.8]	15.0 [9.2, 20.5]^A^	**0.004**
Trombocytes (×10^9^/L) (median [IQR])	198 [142, 282]	225 [161, 294]	206 [147, 269]	0.448
C‐reactive protein (mg/L) (median [IQR])	75 [23, 176]	60 [10, 141]	67 [19, 201]	0.244
Creatinine (umol/L) (median [IQR])	98 [72, 141]^B,C^	114 [90, 190]^A^	136 [91, 206]^A^	**< 0.001**
Bilirubin (umol/L) (median [IQR])	12 [7, 19]	9 [7, 17]	12 [8, 21]	0.248
Scoring systems at triage				
qSOFA (median [IQR])	1 [1, 2]	1 [1, 1]^C^	1 [1, 2]^B^	**0.046**
SOFA (median [IQR])	4 [2, 7]^C^	4 [3, 6]	5 [4, 9]^A^	**0.027**
NEWS (median [IQR])	6 [5, 9]^B^	5 [3, 8]^A,C^	7 [5, 10]^B^	**0.004**
Hemodynamic support therapy				
Prehospital I.V. fluids (i.e., in ambulance) (L) (median [IQR])	0.5 [0.0, 0.5]^C^	0.5 [0.0, 0.5]	0.50 [0.5, 0.5]^A^	0.063
ED I.V. fluids (L) (median [IQR])	0.5 [0.1, 1.5]	0.6 [0.1, 1.3]	1.0 [0.1, 2.0]	0.436
Resuscitation according to 30 mL/kg/3 h (*n* (%))	33 (19)	11 (15)	19 (25)	0.247
Vasopressor therapy (i.e., noradrenaline) < 24 h (*n* (%))	22 (13)	8 (11)	23 (31)	**0.001**

*Note:* The table shows median and interquartile ranges (IQR) between brackets for continuous variables and absolute number and percentages for categorical variables. For continuous variables the Kruskal Wallis test is used to test differences between groups, for categorical variables the Chi‐Squared tests. Significant *p*‐values printed in bold. Pairwise comparisons were subsequently performed using the Wilcoxon rank‐sum test, with *p*‐values adjusted for multiple comparisons using the Bonferroni correction. Letters (A, B, C) above each cluster indicate which other cluster it is significantly different from (*p* < 0.05).

Abbreviations: COPD, chronic obstructive pulmonary disease; GCS, Glasgow Coma Scale; IQR, interquartile range; NEWS, national early warning score; SOFA, sequential organ failure assessment; qSOFA, quick sequential organ failure assessment.

**FIGURE 5:**
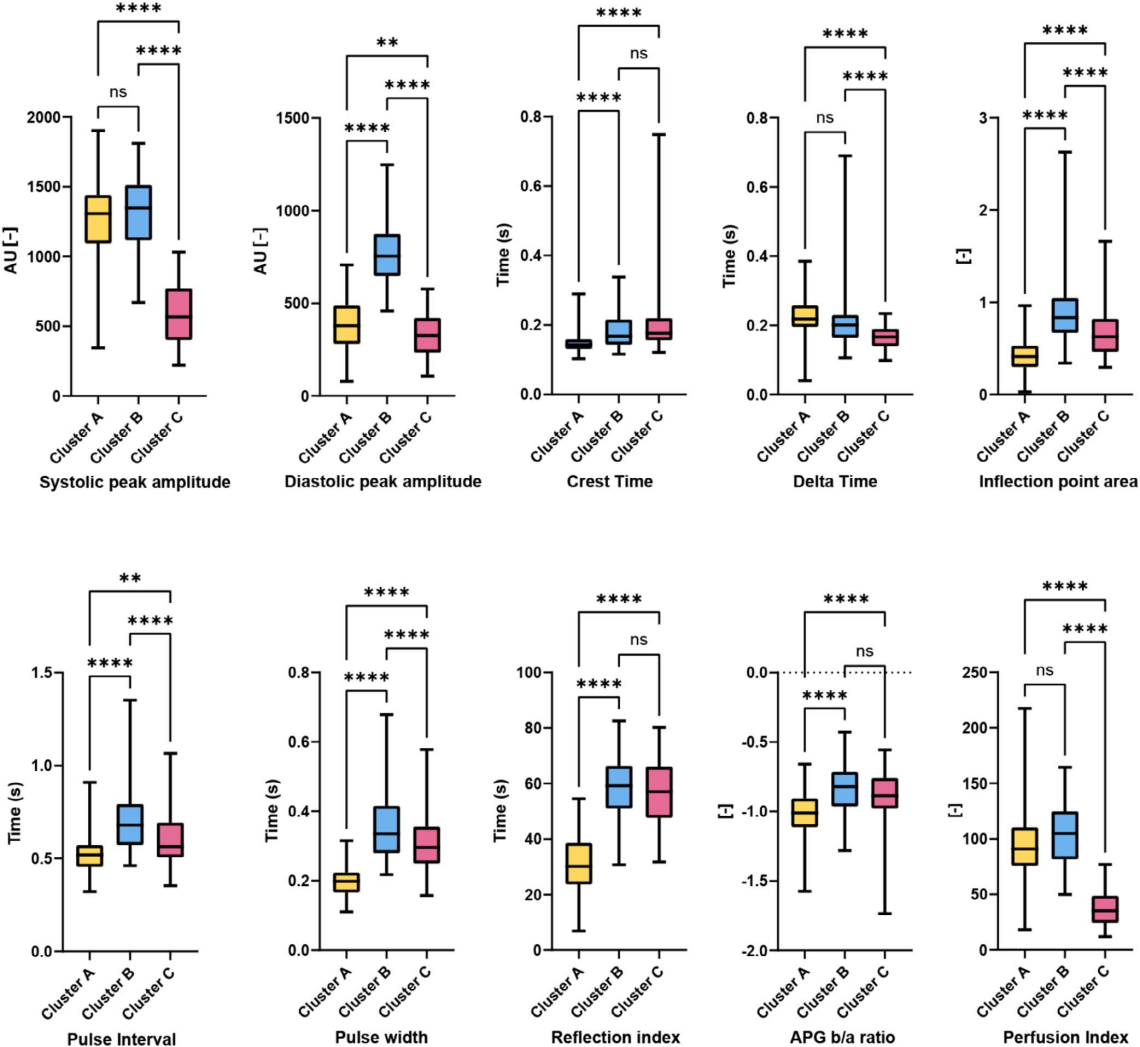
Distribution of PPG‐derived features across PC clusters. The distribution of PPG‐derived features across the identified PC clusters. Clusters on the *x*‐axis, PPG feature value on the y‐axis; ** < 0.01; ***; < 0.001 ****; < 0.001; ns, not significant; AU, arbitrary units; s, seconds; APG, acceleration photoplethysmogram b/a‐ratio.

**FIGURE 6:**
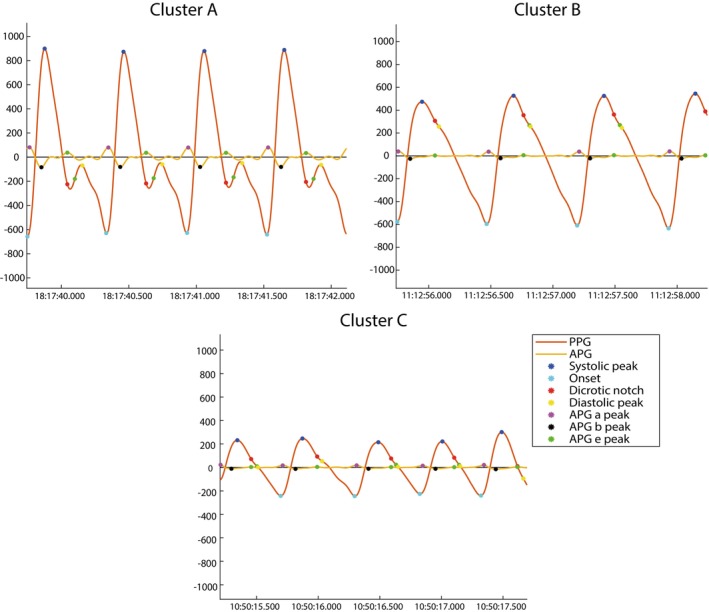
Representative photoplethysmography (PPG) waveforms per PC cluster. Representative PPG waveforms from the patients closest to the centroid of each cluster are shown. These waveforms illustrate the characteristic morphology associated with each cluster, as identified by PCA and *K*‐means clustering of PPG‐derived features during the first 20 min after ED admission. Points of interest (POIs) plotted include the systolic peak, diastolic peak, dicrotic notch, waveform onset, and acceleration plethysmography (APG) components a, b, and e. The features used for clustering, such as systolic peak amplitude, pulse interval, and others, are calculated based on these POIs, reflecting distinct hemodynamic phenotypes and supporting the physiological relevance of the clusters.

To further characterize the hemodynamic differences between clusters, we examined the distribution of individual PPG‐derived features, illustrated in Figure 5. Patients in Cluster A had, on average, the lowest IPA, crest time (CT), RI, PW, and APG b/a ratio compared to the other clusters. Cluster B had the highest DPA and broader PI. Cluster C exhibited the lowest SPA, DPA, DT, and PPI. These features in Cluster C are linked to impaired cardiac output, decreased systemic vascular resistance and disturbed peripheral vasomotor tone, indicating a hemodynamic state more suggestive of predominantly vasodilative shock (Figure 5). To complement these findings, Figure 6 displays representative PPG waveforms from patients closest to each cluster centroid, highlighting morphological patterns that correspond to the identified PPG‐derived features, representing distinct hemodynamic profiles.

### 3.3 PCA Regression Model and Discriminative Power

Multivariable logistic regression was performed using PC1–PC3, which together explained 80.3% of the PPG variance. To adjust for confounders, we included age, gender, and cardiovascular comorbidity (mild: hypertension/diabetes mellitus; moderate: ischemic heart disease; severe: heart failure), as well as pre‐hospital I.V. fluids.

**FIGURE 7:**
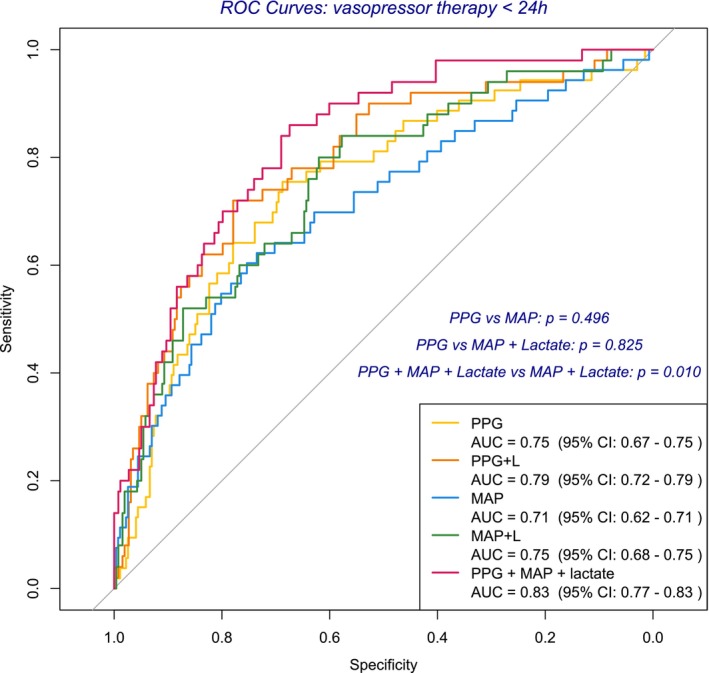
Receiver operating characteristic (ROC) curve and discriminative power of hemodynamic models for vasopressor initiation within 24 h of ED admission. This figure displays the discriminative power of various models integrating PPG, MAP and Lactate in identifying patients who required vasopressor therapy within 24 h of ED admission. The PPG‐only model yielded an AUROC of 0.75, with a sensitivity of 69% and a specificity of 76%. It achieved a positive predictive value (PPV) of 94% and a negative predictive value (NPV) of 32%, resulting in a balanced accuracy of 72% (−2LL = 260.85; *R* ^2^ = 0.178). The PPG + Lactate model improved performance with an AUROC of 0.79, sensitivity of 75%, and specificity of 72%. PPV was 93%, NPV 36%, and balanced accuracy 73% (−2LL = 227.26; *R* ^2^ = 0.352). The MAP‐only model showed a lower AUROC of 0.71, with a sensitivity of 62% and a specificity of 70%. PPV was 91%, NPV 26%, and balanced accuracy 66% (−2LL = 264.84; *R* ^2^ = 0.155). The MAP + Lactate model had an AUROC of 0.75, with sensitivity of 62%, specificity of 80%, PPV of 94%, NPV of 29%, and a balanced accuracy of 71% (−2LL = 253.40; *R* ^2^ = 0.220). The PPG + MAP + Lactate model demonstrated the highest discriminative power with an AUROC of 0.83. It achieved a sensitivity of 67%, specificity of 84%, PPV of 96%, and NPV of 33%, resulting in a balanced accuracy of 76% (−2LL = 227.41; *R* ^2^ = 0.351). Among all models, the PPG + MAP + Lactate model performed best (*p* = 0.010). Notably, the PPG‐only model and the MAP + Lactate model showed comparable performance (*p* = 0.825). PPG, photoplethysmography; MAP, mean arterial pressure; ROC, receiver operating characteristic; AUROC, Area under the ROC.

The PPG‐only model demonstrated moderate discriminative power for identifying patients who received vasopressor therapy within 24 h (AUROC: 0.746), with significant contributions from PC2 and PC3 (*p* < 0.05). Its performance was comparable to the classic MAP‐only model (AUROC: 0.71; *p* = 0.496). The MAP + lactate model showed similar performance (AUROC: 0.75), while adding lactate to PPG improved discrimination (AUROC: 0.79). The combined PPG + MAP + lactate model performed best (AUROC: 0.83), with a sensitivity of 67%, specificity of 84%, positive predictive value (PPV) of 96%, and negative predictive value (NPV) of 33%, significantly outperforming both the PPG‐only and MAP + lactate models (*p* = 0.010), which did not differ from each other (*p* = 0.824). Performance metrics for all models are detailed in Figure 7.

The combined model also showed moderate discriminative power for secondary endpoints, including patients who required higher‐volume fluid resuscitation (> 30 mL/kg, AUROC: 0.73) and were admitted to the ICU within 48 h (AUROC: 0.77). The discriminative power for in‐hospital mortality was good (AUROC: 0.86). Receiver‐operating characteristic (ROC) curves for secondary endpoints and corresponding performance metrics are presented in Figure S2.

Internal validation with a separate 2024 cohort was also performed, as outlined in Table S3 which includes study population characteristics, and Figure S3, where sensitivity, specificity, PPV, NPV, and accuracy metrics highlight the model's robustness across cohorts. In the internal validation cohort, the combined model again had the highest AUROC but did not significantly outperform the MAP + lactate model, likely due to the smaller sample size and imbalanced outcome distribution (5% vasopressor use, Table S3). Importantly, it did not perform worse, supporting the robustness and added value of PPG‐derived features. Notably, the PPG‐only model performed as well as the MAP + lactate model (Figure S3), reinforcing the potential of PPG to capture peripheral perfusion dynamics in septic shock and serve as a non‐invasive alternative to established clinical parameters.

## 4 Discussion

### 4.1 Main Findings

This study investigated whether PPG‐derived features measured during the first 20 min of ED admission could identify early septic shock subphenotypes, and could help identify patients with predominant vasodilatory shock requiring vasopressor therapy within 24 h. First, unsupervised K‐means clustering using PPG components identified three patient subphenotypes with partially overlapping characteristics but distinct clinical and hemodynamic profiles, including differences in age, hypertension, blood pressure, lactate, and creatinine. These differences underscore the ability of PPG‐based clustering to capture clinically meaningful variations among patients. Cluster C comprised patients in septic shock, characterized by worsened vital signs, a greater need for hemodynamic support therapy, ICU admission, and increased mortality. Additionally, patients with absent or poor‐quality PPG signals show characteristics similar to Cluster C, suggesting underlying hemodynamic compromise and supporting prior findings on the prognostic value of absent pulse oximeter signals in prehospital care [35]. In contrast, Clusters A and B consisted of patients with suspected sepsis and early hemodynamic instability, who received similar volumes of I.V. fluids but experienced better outcomes than Cluster C. While these clusters are not sharply defined, they reflect a spectrum of hemodynamic changes in sepsis, from a more normal pattern to a vasodilatory state with or without concurrent hypovolemia, suggesting that non‐invasive PPG could offer valuable insights for tailoring sepsis management based on a patient's specific hemodynamic profile.

Second, the supervised multivariable analysis showed that a discriminative model based solely on PPG‐derived features demonstrated moderate accuracy in identifying patients who required vasopressor therapy within 24 h. However, a model combining PPG with MAP proved to be more accurate in identifying patients who required vasopressors, suggesting that PPG provides additional valuable insights beyond MAP alone. MAP reflects the interplay between cardiac output and systemic vascular resistance, while PPG can detect peripheral pathophysiological changes, such as vasodilation. This suggests that integrating PPG could help recognize patients with early septic shock characterized by a predominant vasodilatory component who might benefit from vasopressor therapy early, thereby allowing for more tailored hemodynamic resuscitation and improving sepsis outcomes.

### 4.2 Pathophysiological Explanation

PPG‐derived features can be grouped via PCA into distinct components that further refine our understanding of the hemodynamic state. For instance, PC1, primarily characterized by PW and RI, is associated with arterial compliance [26, 27]. PC2, encompassing features such as PPI and SPA, reflects peripheral perfusion influenced by both CO and SVR [28, 34, 36]. PC3, reflecting DPA and DT, provides insight into vasomotor tone alterations such as vasodilation, where fluid resuscitation is unlikely to be effective and early vasopressor initiation may be preferable [28, 37, 38]. This physiological relevance underscores the potential of PPG as a clinical decision support tool that continuously and non‐invasively capturing dynamic hemodynamic changes through peripheral perfusion measurements at the fingertip.

Although MAP provides insight into hemodynamic status, it primarily reflects central circulatory function and does not fully capture peripheral changes [39, 40]. Similarly, lactate levels are an important biomarker for hypoperfusion and organ dysfunction in sepsis, but they do not offer a comprehensive view of cardiovascular dynamics [41, 42]. In fact, a recent study showed that continued resuscitation on lactate levels in patients with normal peripheral perfusion might even be harmful [42]. Moreover, their findings suggest that a resuscitation strategy targeting peripheral perfusion status may be superior to one based solely on lactate levels [43]. In this context, PPG provides a continuous, non‐invasive method to assess peripheral perfusion and hemodynamic changes, thereby potentially serving as a valuable tool to guide personalized hemodynamic resuscitation in sepsis. Integrating PPG‐derived components with MAP and lactate may thus offer a more comprehensive hemodynamic profile, potentially improving the ability to recognise patients with a predominant vasodilatory profile requiring earlier vasopressor therapy, and to guide tailored management strategies in sepsis.

### 4.3 Clinical Relevance

This study emphasizes the need for a personalized approach to hemodynamic resuscitation in sepsis. PPG can recognize dynamic hemodynamic changes in sepsis, allowing the identification of hemodynamic profiles and distinguishing between different subphenotypes of septic shock. Sepsis often presents as a combination of hypovolaemic and distributive shock, requiring a balance of therapeutic strategies: vasopressor therapy for the vasodilatory component and volume resuscitation to correct hypovolaemia [24]. While a personalized approach is essential, traditional measures such as MAP and lactate have limitations in assessing the full spectrum of circulatory dysfunction [20, 42, 44]. PPG, however, offers additional real‐time, non‐invasive insights into cardiovascular status in sepsis [45, 46]. An additional advantage is its ability to provide continuous monitoring through wearable devices, which can be used not only in the ED but also in prehospital care, general practice, and home care settings [47]. This capability supports PPG as a promising clinical decision support tool to facilitate the early identification of predominant shock type and guide clinical decision‐making, ensuring the selection of the most appropriate therapeutic intervention for the individual, and optimizing clinical outcomes in sepsis management.

### 4.4 Future Implications

PPG offers a non‐invasive, continuous approach to monitor cardiovascular dynamics, and holds promise as a clinical decision support tool for personalized hemodynamic resuscitation in sepsis. This proof‐of‐principle study establishes an important foundation but further steps are necessary to advance clinical implementation. Future research should first perform external validation to confirm that PPG reliably captures cardiovascular characteristics across diverse patient populations [48, 49]. Second, model development should focus on creating clinically interpretable algorithms by selecting important features, defining actionable thresholds, and exploring computational methods suitable for real‐time decision support [49, 50, 51]. Third, clinical implementation requires optimizing and automating the signal processing pipeline for near real‐time application at bedside, potentially extending to wearable devices [51, 52, 53]. Fourth, clinical trial enrichment should evaluate the role of PPG in predictive and prognostic enrichment [54, 55], particularly to guide early vasopressor initiation in patients with predominant vasodilatory septic shock. Finally, broadened application should investigate the additional value of PPG in assessing fluid responsiveness and monitoring treatment effects, as previous studies have linked the perfusion index to fluid responsiveness in ICU patients with septic shock [56, 57]. In summary, future work should focus on improving the technique and methodology, performing robust external validation, and developing practical real‐time bedside tools with actionable thresholds for integration into sepsis resuscitation protocols.

### 4.5 Limitations

Several limitations should be acknowledged in this study. Firstly, it was conducted in a single tertiary care centre. However, the large rural/urban catchment area ensures that the case mix is representative of both general and academic teaching hospitals. As an observational study, missing data posed a challenge. Nonetheless, essential information was manually verified to minimize its impact. While missing data is an inherent limitation in real‐world studies, it also increases the likelihood of successful implementation in clinical practice. Additionally, to minimize technical artifacts, a strict SQI algorithm (0.8) was applied, resulting in the exclusion of some data from the analysis. This approach ensured the inclusion of high‐quality data, even for patients with low peripheral perfusion due to septic shock. Only 7.1% of the patients were excluded due to absent or poor quality, in the first 20 min, and this quality control procedure is not expected to introduce bias. Notably, a subgroup analysis showed that patients without usable PPG signals had clinical characteristics similar to those in the septic shock subphenotype, indicating that poor‐quality signals may also reflect hemodynamic compromise.

## 5 Conclusion

In conclusion, this proof‐of‐principle study demonstrated that early, non‐invasive photoplethysmography (PPG) measurement during emergency department triage can identify septic shock subphenotypes and moderately distinguish patients who received subsequent vasopressor therapy, likely due to a vasodilatory profile, thus providing additional value beyond classical parameters such as mean arterial pressure and lactate. By early identification of vasodilatory shock, with or without a hypovolemic component, through the continuous assessment of peripheral perfusion and hemodynamic changes at the fingertip, PPG may help identify patients who might benefit from early initiation of vasopressor therapy as a part of a personalized hemodynamic resuscitation strategy. This approach could support clinical decision making by carefully balancing the choice between higher‐volume fluid resuscitation and early initiation of vasopressor therapy, ultimately optimizing sepsis management and potentially improving patient outcomes.

## Author Contributions

**Sanne Ter Horst:** conceptualization, methodology, software, formal analysis, investigation, writing ‑ original draft, visualization, project administration. **Anna D. Schoonhoven:** methodology, software, investigation, writing ‑ review and editing. **Raymond J. van Wijk:** methodology, software, investigation, writing ‑ review and editing. **Rick Weitering:** investigation, data curation, writing ‑ review and editing. **Sanne W. van Loon:** investigation, data curation, writing ‑ review and editing. **Jan C. ter Maaten:** methodology, conceptualization, writing ‑ review and editing, supervision. **Hjalmar R. Bouma:** conceptualization, methodology, validation, writing ‑ review and editing, supervision, project administration.

## Conflicts of Interest

The authors declare no conflicts of interest.

## Supporting information


**Figure S1:** Correlation matrix among PPG features and PCs. A correlation matrix illustrating the relationships between individual PPG features and the principal components. Spearman correlation coefficients (*ρ*) are determined by the Spearman correlation test. Numbers represent significant correlation coefficients (*p* < 0.05). Among the PPG features, strong correlations were observed between SPA and PPI (*ρ* = 0.98), and between DT and RI (*ρ* = 0.81). PI was correlated with PW (*ρ* = 0.74), while PW showed a strong correlation with RI (*ρ* = 0.81), and RI was also associated with IPA (*ρ* = 0.81). Regarding the principal components, PC1 was strongly correlated with IPA (*ρ* = 0.80), PI (*ρ* = 0.72), PW (*ρ* = 0.95), and RI (*ρ* = 0.92); PC2 with SPA (*ρ* = 0.94) and PPI (*ρ* = 0.96); and PC3 with DPA (*ρ* = 0.56) and DT (*ρ* = 0.59).

**Figure S2:** Receiver operating characteristic (ROC) curves illustrating the discriminative power of secondary endpoints, including administration of I.V. fluid resuscitation > 30 mL/kg, ICU admission < 48 h, and in‐hospital mortality < 48 h. The logistic regression model incorporating PPG's Principal Components 1–3, age, gender, and CVD demonstrated moderate discriminative power for I.V. fluid administration > 30 mL/kg during the ED stay, with an AUROC of 0.67 (−2LL: 298.32, *R* ^2^: 0.123). Including MAP and lactate resulted in an AUROC to 0.73 (−2LL: 287.50, *R* ^2^: 0.183), while a model with only MAP and lactate achieved an AUROC of 0.70 (−2LL: 205.60, *R* ^2^: 0.140). For ICU admission within 48 h, the basic PPG model showed an AUROC of 0.73 (−2LL: 319.62, *R* ^2^: 0.229). Incorporating PPG, MAP, and lactate increased the AUROC to 0.77 (−2LL: 305.34, *R* ^2^: 0.289), whereas a model with only MAP and lactate resulted in a comparable AUROC of 0.68 (−2LL: 345.47, *R* ^2^: 0.111). The discriminative power for in‐hospital mortality within 48 h showed an AUROC of 0.80 (−2LL: 95.93, *R* ^2^: 0.289). Including PPG, MAP, and lactate resulted in an AUROC 0.86 (−2LL: 85.56, *R* ^2^: 0.411), while a model with only MAP and lactate achieved an AUROC of 0.83 (−2LL: 91.45, *R* ^2^: 0.344).

**Figure S3:** Receiver‐operating characteristic (ROC) curves for the discriminative power of primary outcome, that is, vasopressor therapy within 24 h in the internal 2024 validation cohort. The logistic regression model incorporates PPG's Principal Components 1–3, age, gender, cardiovascular disease (CVD), and pre‐hospital intravenous fluids. The model achieved an AUROC of 0.74, with a sensitivity of 84%, specificity of 60%, a positive predictive value of 98%, and a negative predictive value of 15%. The corrected balanced accuracy was 72%. Model performance metrics: −2LL = 72.42, *R* ^2^ = 0.300.

**Data S1:** Supporting Information.

## Data Availability

The data that support the findings of this study are available on reasonable request from the corresponding author. The data are not publicly available due to privacy or ethical restrictions.
