# Supplementary figures and images for: A Novel Approach to Early Personalized Hemodynamic Resuscitation: Non‐Invasive Peripheral Photoplethysmography for Identifying Predominant Vasodilatory Shock in Sepsis
Source: Acta Anaesthesiol Scand. 2025 Sep 9;69(9):e70119. doi: 10.1111/aas.70119 (PMC12418295; doi:10.1111/aas.70119)

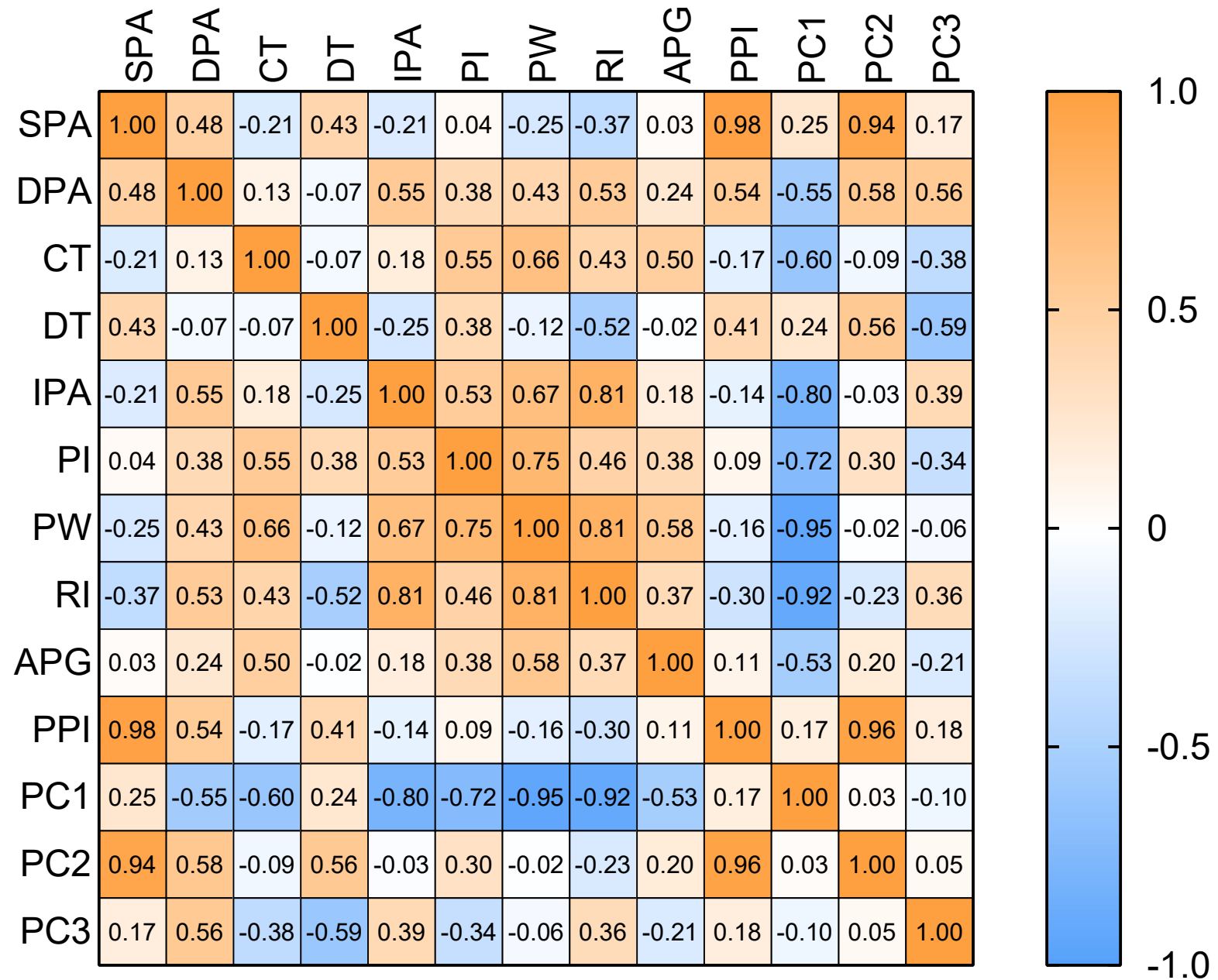

Supplement: Supplementary file 1 — Figure S1: Correlation matrix among PPG features and PCs. A correlation matrix illustrating the relationships between individual PPG features and the principal components. Spearman correlation coefficients (ρ) are determined by the Spearman correlation test. Numbers represent significant correlation coefficients (p < 0.05). Among the PPG features, strong correlations were observed between SPA and PPI (ρ = 0.98), and between DT and RI (ρ = 0.81). PI was correlated with PW (ρ = 0.74), while PW showed a strong correlation with RI (ρ = 0.81), and RI was also associated with IPA (ρ = 0.81). Regarding the principal components, PC1 was strongly correlated with IPA (ρ = 0.80), PI (ρ = 0.72), PW (ρ = 0.95), and RI (ρ = 0.92); PC2 with SPA (ρ = 0.94) and PPI (ρ = 0.96); and PC3 with DPA (ρ = 0.56) and DT (ρ = 0.59). [file AAS-69-0-s002.pdf]

*ROC Curves Validation cohort: Vasopressor therapy < 24h*

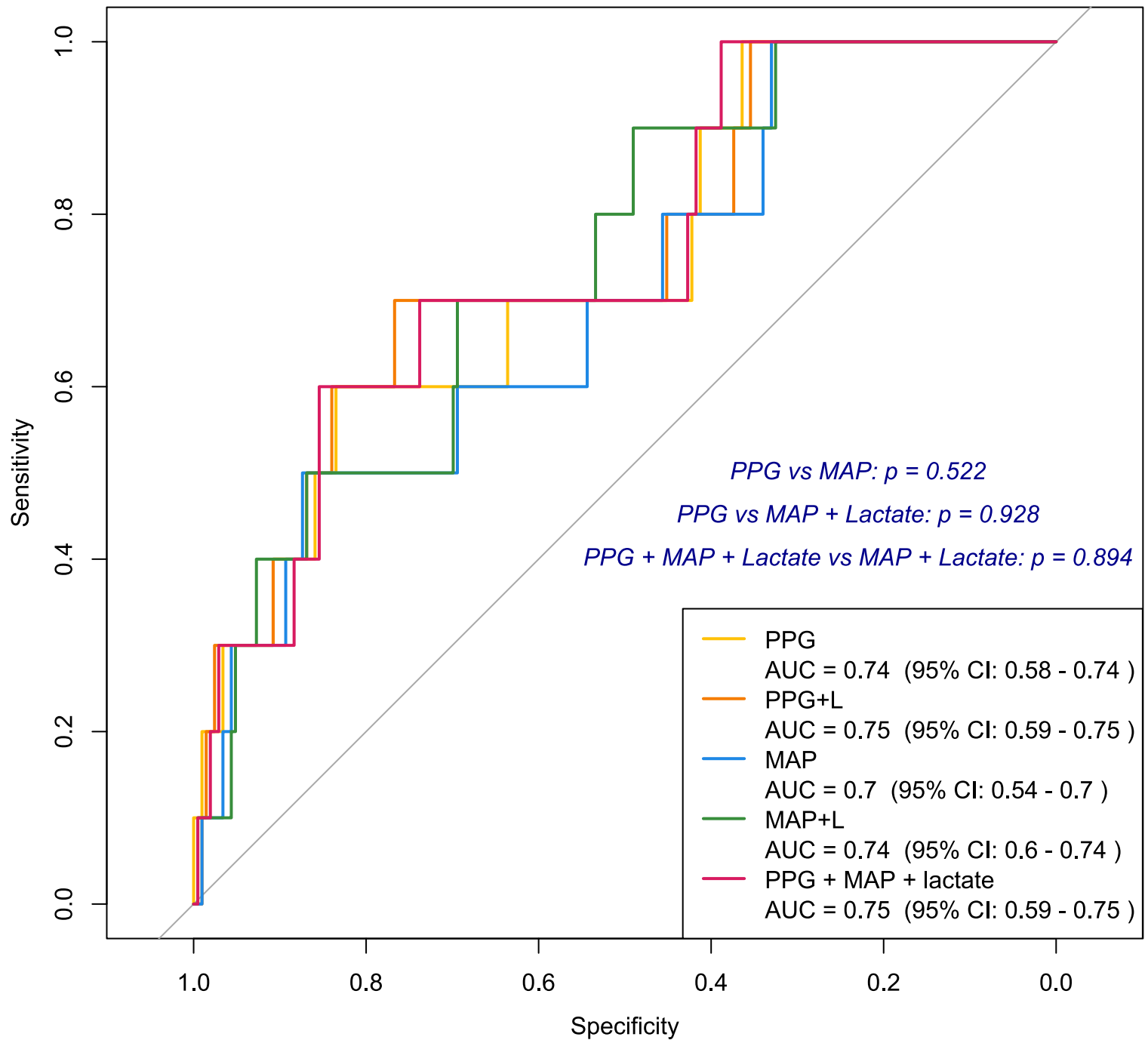

Supplement: Supplementary file 3 — Figure S3: Receiver‐operating characteristic (ROC) curves for the discriminative power of primary outcome, that is, vasopressor therapy within 24 h in the internal 2024 validation cohort. The logistic regression model incorporates PPG's Principal Components 1–3, age, gender, cardiovascular disease (CVD), and pre‐hospital intravenous fluids. The model achieved an AUROC of 0.74, with a sensitivity of 84%, specificity of 60%, a positive predictive value of 98%, and a negative predictive value of 15%. The corrected balanced accuracy was 72%. Model performance metrics: −2LL = 72.42, R 2 = 0.300. [file AAS-69-0-s004.pdf]
