# Supplementary material for: A Novel Approach to Early Personalized Hemodynamic Resuscitation: Non‐Invasive Peripheral Photoplethysmography for Identifying Predominant Vasodilatory Shock in Sepsis
Source: Acta Anaesthesiol Scand. 2025 Sep 9;69(9):e70119. doi: 10.1111/aas.70119 (PMC12418295; doi:10.1111/aas.70119)
Supplement: Supplementary file 2 — Figure S2: Receiver operating characteristic (ROC) curves illustrating the discriminative power of secondary endpoints, including administration of I.V. fluid resuscitation > 30 mL/kg, ICU admission < 48 h, and in‐hospital mortality < 48 h. The logistic regression model incorporating PPG's Principal Components 1–3, age, gender, and CVD demonstrated moderate discriminative power for I.V. fluid administration > 30 mL/kg during the ED stay, with an AUROC of 0.67 (−2LL: 298.32, R 2: 0.123). Including MAP and lactate resulted in an AUROC to 0.73 (−2LL: 287.50, R 2: 0.183), while a model with only MAP and lactate achieved an AUROC of 0.70 (−2LL: 205.60, R 2: 0.140). For ICU admission within 48 h, the basic PPG model showed an AUROC of 0.73 (−2LL: 319.62, R 2: 0.229). Incorporating PPG, MAP, and lactate increased the AUROC to 0.77 (−2LL: 305.34, R 2: 0.289), whereas a model with only MAP and lactate resulted in a comparable AUROC of 0.68 (−2LL: 345.47, R 2: 0.111). The discriminative power for in‐hospital mortality within 48 h showed an AUROC of 0.80 (−2LL: 95.93, R 2: 0.289). Including PPG, MAP, and lactate resulted in an AUROC 0.86 (−2LL: 85.56, R 2: 0.411), while a model with only MAP and lactate achieved an AUROC of 0.83 (−2LL: 91.45, R 2: 0.344). [file AAS-69-0-s001.pdf]

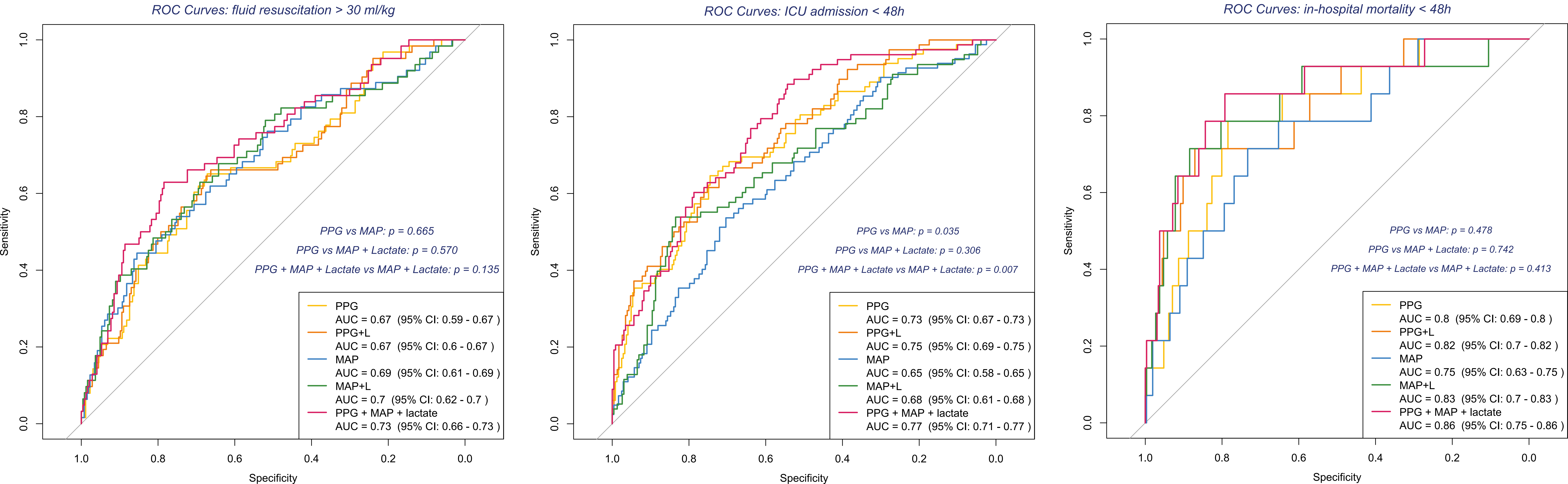
ROC Curves: fluid resuscitation > 30 ml/kg
Sensitivity
Specificity
PPG vs MAP: p = 0.665
PPG vs MAP + Lactate: p = 0.570
PPG + MAP + Lactate vs MAP + Lactate: p = 0.135
PPG
AUC = 0.67 (95% CI: 0.59 - 0.67)
PPG+L
AUC = 0.67 (95% CI: 0.6 - 0.67)
MAP
AUC = 0.69 (95% CI: 0.61 - 0.69)
MAP+L
AUC = 0.7 (95% CI: 0.62 - 0.7)
PPG + MAP + lactate
AUC = 0.73 (95% CI: 0.66 - 0.73)
ROC Curves: ICU admission < 48h
Sensitivity
Specificity
PPG vs MAP: p = 0.035
PPG vs MAP + Lactate: p = 0.306
PPG + MAP + Lactate vs MAP + Lactate: p = 0.007
PPG
AUC = 0.73 (95% CI: 0.67 - 0.73)
PPG+L
AUC = 0.75 (95% CI: 0.69 - 0.75)
MAP
AUC = 0.65 (95% CI: 0.58 - 0.65)
MAP+L
AUC = 0.68 (95% CI: 0.61 - 0.68)
PPG + MAP + lactate
AUC = 0.77 (95% CI: 0.71 - 0.77)
ROC Curves: in-hospital mortality < 48h
Sensitivity
Specificity
PPG vs MAP: p = 0.478
PPG vs MAP + Lactate: p = 0.742
PPG + MAP + Lactate vs MAP + Lactate: p = 0.413
PPG
AUC = 0.8 (95% CI: 0.69 - 0.8)
PPG+L
AUC = 0.82 (95% CI: 0.7 - 0.82)
MAP
AUC = 0.75 (95% CI: 0.63 - 0.75)
MAP+L
AUC = 0.83 (95% CI: 0.7 - 0.83)
PPG + MAP + lactate
AUC = 0.86 (95% CI: 0.75 - 0.86)
