# Supplementary material for: A Novel Approach to Early Personalized Hemodynamic Resuscitation: Non‐Invasive Peripheral Photoplethysmography for Identifying Predominant Vasodilatory Shock in Sepsis
Source: Acta Anaesthesiol Scand. 2025 Sep 9;69(9):e70119. doi: 10.1111/aas.70119 (PMC12418295; doi:10.1111/aas.70119)
Supplement: Supplementary file 4 — Data S1: Supporting Information. [file AAS-69-0-s003.docx]

# **Supplemental tables**

**Supplemental Table W: Description of photoplethysmography-derived features**

| **Photoplethysmography feature** | **Description** |
| --- | --- |
| **Systolic peak amplitude (SPA)** | The SPA is the height of the first PPG waveform peak, and it is related to stroke volume (28,32). Multiple factors influence the SPA. Pulsatile changes in blood volume and peripheral vasoconstriction or vasodilation affect the height of the peak. For example, decreased blood volume pulsation with peripheral vasoconstriction causes a lower systolic peak amplitude  (28). |
| **Diastolic peak amplitude (DPA)** | The DPA is the height of the second PPG waveform peak. It is the result of the reflected blood pressure from small blood vessels and this blood pressure arises from the systolic pressure pulse (27,33). Changes in diastolic peak height are caused by total peripheral resistance and/or arterial compliance (28). |
| **Pulse width (PW)** | This feature is the pulse width defined at half the height of SPA. It is indicated that the pulse width has a correlation with the systemic vascular resistance (28,32,33). |
| **Pulse interval (PI)** | The pulse interval is the distance between the beginning and the end of the PPG waveform (27). The pulse interval of PPG is strongly correlated with the R-R interval in electrocardiogram (ECG) (28). |
| **Inflection point area (IPA)** | The area under the PPG waveform is divided by the inflection point at the dicrotic notch. The IPA ratio is the division of these two different areas (Area 2/Area 1). This feature is related to total peripheral resistance (28). |
| **Reflection index (RI)** | The RI is calculated by dividing the DPA by SPA and multiplying it by 100%. RI measures the reflected wave coming from the periphery to the centre. Changes in RI are associated with changes in elastic arteries compliance (28,33). |
| **Crest time (CT)** | The CT is the time it takes from the foot of the PPG waveform to its first peak. Crest time is related to pulse wave velocity. In literature it is found to be useful for cardiovascular disease classification (28,33). For example, it is longer in vascular disease or hypertension patients (27). |
| **Delta time (DT)** | DT is the time taken for the blood ejected from the heart to pass to the peripheral blood vessel. In literature it is found to be one of the best features for accurate classification of cardiovascular disease (28,32). |
| **APG - b/a-ratio (APG)** | The APG b/a-ratio is proportional to the stiffness of blood vessels and increases with age. A higher b/a-ratio represents increased arterial stiffness (32). The magnitude of b/a of the APG is related to the distensibility of the peripheral artery and suggests that the b/a-ratio is a useful non-invasive index of altered arterial distensibility (28). |
| **Perfusion index (PPI)** | The perfusion index (PPI), derived from the PPG signal, reflects the ratio of pulsatile to non-pulsatile light absorption and is influenced by vascular tone and stroke volume (34). The PPI indicates vasodilation (higher PPI) or vasoconstriction (lower PPI) in peripheral vessels. In septic shock, however, peripheral vasodilation can happen even with poor tissue perfusion, causing a lower PPI despite impaired circulation (34). |

Abbreviations: SPA: systolic peak amplitude; DPA: diastolic peak amplitude; PW: pulse width; PI: pulse interval; IPA: inflection point area; RI: reflection index; CT: crest time; DT: delta time; APG: acceleration plethysmogram PPI: Photoplethysmography perfusion index;

**Supplemental Table Y.** **Comparison of clinical characteristics between cluster C and the absent or low quality PPG signal group**

| **Characteristics** | **Cluster C group,**  **N = 75** | **Absent or low quality PPG group,**  **N = 28** | **P value** |
| --- | --- | --- | --- |
| **Demographics** |  |  |  |
| Female (n (%)) | 26 (35) | 10 (36) | 1.000 |
| Age (median [IQR]) | 70 [57, 77] | 64 [53, 73] | 0.197 |
| **Co-morbidities** |  |  |  |
| Charlson Comorbidity Index (median [IQR]) | 4 [3, 7] | 3 [2, 5] | 0.164 |
| Ischemic heart disease (n (%)) | 9 (12) | 1 (4) | 0.362 |
| Heart failure (n (%)) | 10 (13) | 6 (21) | 0.482 |
| Hypertension (n (%)) | 35 (47) | 5 (18) | **0.015** |
| Diabetes (n (%)) | 17 (23) | 3 (11) | 0.278 |
| **Vital parameters at triage** |  |  |  |
| Systolic blood pressure [mmHg] (median [IQR]) | 99 [84, 115] | 106 [89, 117] | 0.324 |
| Diastolic blood pressure [mmHg] (median [IQR]) | 61 [51, 76] | 72 [59, 86] | 0.068 |
| Mean arterial blood pressure [mmHg] (median [IQR]) | 74 [63, 87] | 83 [70, 96] | 0.120 |
| Heart rate [beats/min] (median [IQR]) | 111 [95, 121] | 112 [96, 125] | 0.900 |
| Respiratory frequency [/min] (median [IQR]) | 23 [20, 27] | 24 [19, 27] | 0.947 |
| Oxygen Saturation (SpO2) [%] (median [IQR]) | 97 [94, 99] | 96 [91, 99] | 0.915 |
| Temperature (ºC) (median [IQR]) | 36.6 [36.1, 37.4] | 36.6 [36.0, 37.4] | 0.849 |
| GCS (median [IQR]) | 15 [15, 15] | 15 [14, 15] | 0.062 |
| **Lab values at triage** |  |  |  |
| Blood gas lactate (mmol/L) (median [IQR]) | 3.8 [2.8, 5.2] | 3.2 [2.6, 4.6] | 0.714 |
| **Scoring systems at triage** |  |  |  |
| qSOFA (median [IQR]) | 1 [1, 2] | 1 [1, 2] | 0.844 |
| SOFA (median [IQR]) | 5 [4, 9] | 6 [3, 10] | 0.793 |
| NEWS (median [IQR]) | 7 [5, 10] | 7 [5, 9] | 0.896 |
| **Hemodynamic support therapy** |  |  |  |
| Prehospital I.V. fluids (i.e. in ambulance) (L) (median [IQR]) | 0.5 [0.5, 0.5] | 0.5 [0.0, 0.5] | 0.487 |
| ED I.V. fluids (L) (median [IQR]) | 1.0 [0.1, 2.0] | 1.3 [0.0, 1.6] | 0.750 |
| Resuscitation according to 30ml/kg/3h (n(%)) | 19 (25) | 6 (21) | 0.878 |
| Vasopressor therapy (i.e. noradrenaline) <24h (n(%)) | 23 (31) | 9 (32) | 1.000 |
| **Clinical deterioration outcomes** |  |  |  |
| ICU admission < 48h (n(%)) | 33 (44) | 12 (43) | 1.000 |
| In-hospital mortality <48h | 8 (11) | 2 (7) | 0.870 |

This table compares patients in PPG-derived Cluster C with those in whom absent or low quality PPG signal was available during the first 20 minutes. Absence of a signal was due either to no measurement or to low signal quality as determined by the Signal Quality Index (SQI). Continuous variables are presented as medians with interquartile ranges (IQR) in parentheses; categorical variables as absolute numbers with percentages. Group differences were tested using the Mann–Whitney U test for continuous variables and the Chi-squared test for categorical variables. Statistically significant p-values are shown in bold. No significant differences in hemodynamic status or clinical endpoints were observed between groups, suggesting that absence of a PPG signal may itself hold prognostic value for worse clinical outcomes and may indicate the potential benefit of early vasopressor use. IQR: interquartile range; GCS: Glasgow Coma Scale; SOFA: Sequential Organ Failure Assessment; qSOFA: quick SOFA; NEWS: National Early Warning Score.

**Supplemental Table X.** **Comparison of clinical characteristics and PPG-derived features between patients with and without confirmed infection**

| **Characteristics** | **Infection group*,**  **N = 242** | **No infection group*,**  **N = 83** | **P value** |
| --- | --- | --- | --- |
| **Demographics** |  |  |  |
| Female (n (%)) | 100 (41) | 32 (39) | 0.754 |
| Age (median [IQR]) | 63 [52, 73] | 60 [48, 71] | 0.473 |
| **Co-morbidities** |  |  |  |
| Charlson Comorbidity Index (median [IQR]) | 4 [2, 6] | 3 [1, 5] | 0.164 |
| **Vital parameters at triage** |  |  |  |
| Systolic blood pressure [mmHg] (median [IQR]) | 105 [90, 120] | 104 [92, 123] | 0.773 |
| Diastolic blood pressure [mmHg] (median [IQR]) | 65 [53, 78] | 65 [54, 79] | 0.929 |
| Mean arterial blood pressure [mmHg] (median [IQR]) | 80 [66, 91] | 80 [64, 93] | 0.848 |
| Heart rate [beats/min] (median [IQR]) | 114 [97, 128] | 110 [94, 122] | 0.189 |
| **PPG derived features** |  |  |  |
| Systolic peak amplitude (median [IQR]) | 1190 [885, 1418] | 1141 [807, 1398] | 0.398 |
| Diastolic peak amplitude (median [IQR]) | 405 [298, 579] | 459 [320, 614] | 0.173 |
| Crest time (median [IQR]) | 0.15 [0.14, 0.18] | 0.16 [0.14, 0.20] | 0.192 |
| Delta time (median [IQR]) | 0.20 [0.17, 0.23] | 0.21 [0.17, 0.24] | 0.976 |
| Inflection point area (median [IQR]) | 0.51 [0.35, 0.72] | 0.61 [0.42, 0.82] | **0.026** |
| Pulse interval (median [IQR]) | 0.55 [0.48, 0.64] | 0.60 [0.51, 0.70] | **0.021** |
| Pulse width (median [IQR]) | 0.23 [0.19, 0.29] | 0.27 [0.21, 0.35] | **0.005** |
| Reflection index (median [IQR]) | 40.1 [27.9, 54.5] | 44.9 [33.9, 60.7] | **0.024** |
| APG b/a ratio (median [IQR]) | -0.94 [-1.07, -0.83] | -0.92 [-1.08, -0.76] | 0.532 |
| Perfusion index (median [IQR]) | 83.0 [57.2, 110.6] | 82.0 [53.6, 104.3] | 0.451 |
| **Lab values at triage** |  |  |  |
| Blood gas lactate (mmol/L) (median [IQR]) | 2.6 [1.9, 3.7] | 4.1 [2.3, 6.4] | **<0.001** |
| **Hemodynamic support therapy** |  |  |  |
| Prehospital i.v. fluids (i.e. in ambulance) (L) (median [IQR]) | 0.5 [0.0, 0.5] | 0.5 [0.0, 0.5] | 0.617 |
| Emergency department i.v. fluids (L) (median [IQR]) | 1.0 [0.2, 2.0] | 0.5 [0.0, 1.0] | **<0.001** |
| Resuscitation according to 30ml/kg/3h (n(%)) | 55 (23) | 8 (10) | **0.015** |
| Vasopressor therapy (i.e. noradrenaline) <24h (n(%)) | 40 (17) | 13 (16) | 0.990 |
| **Clinical deterioration outcomes** |  |  |  |
| ICU admission < 48h (n(%)) | 57 (23.6) | 25 (30.1) | 0.297 |
| In-hospital mortality <48h | 10 (4.1) | 4 (4.8) | 1.000 |

*This table presents a comparison between patients with confirmed infection based on a structured post-hoc adjudication process by independent experts, and those in whom infection was not confirmed. Patients were originally included based on clinical suspicion of infection at ED presentation. Continuous variables are reported as medians with interquartile ranges (IQR) in parentheses, and categorical variables as absolute numbers with percentages. Differences between groups were assessed using the Mann–Whitney U test for continuous variables and the Chi-squared test for categorical variables. Statistically significant p-values are indicated in bold. Interestingly, several PPG-derived features, IPA, PW, PI, and RI, differed between groups. PW, RI, and IPA, reflecting arterial compliance and systemic vascular resistance, were lower in the confirmed infection group, suggesting increased vasodilation. PI was shorter in the infection group, consistent with tachycardia. Moreover, lactate levels were higher in the no-infection group, potentially indicating alternative causes of hyperlactatemia (e.g., diabetic ketoacidosis). Despite receiving less intravenous fluid, the no-infection group did not exhibit worse clinical outcomes. IQR: interquartile range; GCS: Glasgow Coma Scale; SOFA: Sequential Organ Failure Assessment; qSOFA: quick SOFA; NEWS: National Early Warning Score; IPA: Inflection point area; PW – Pulse Width; PI – Pulse Interval; RI – Reflection Index.

**Supplemental Table Z: Characteristics of internal validation cohort 2024**

| **Characteristics of the study cohort** | **N = 226** |
| --- | --- |
|  |  |
| **Demographics** |  |
| Female (n (%)) | 97 (43) |
| Age (median [IQR]) | 62.00 [51, 72] |
| **Co-morbidities** |  |
| Charlson Comorbidity Index (median [IQR]) | 4 [2, 6] |
| Hypertension (n (%)) | 67 (30) |
| Ischemic heart disease (n (%)) | 25 (11) |
| Heart failure (n (%)) | 21 (9) |
| Diabetes (n (%)) | 48 (21) |
| **Scoring systems at ED triage** |  |
| SOFA (median [IQR]) | 3 [2, 5] |
| Hemodynamic support therapy |  |
| Vasopressor therapy (i.e. noradrenaline) <24h (n(%)) | 11 (5) |
| **Clinical deterioration outcomes** |  |
| ICU admission < 48h (n(%)) | 22 (10) |
| In-hospital mortality <48h | 50 (22) |

The table shows median and interquartile ranges (IQR) between brackets for continuous variables and absolute number and percentages for categorical variables. IQR: Interquartile range; COPD: Chronic Obstructive Pulmonary Disease; GCS: Glasgow Coma Scale; SOFA: Sequential Organ Failure Assessment; qSOFA: quick Sequential Organ Failure Assessment; NEWS: National Early Warning Score.
